# An automated shotgun lipidomics platform for high throughput, comprehensive, and quantitative analysis of blood plasma intact lipids

**DOI:** 10.1002/ejlt.201500145

**Published:** 2015-07-20

**Authors:** Michal A Surma, Ronny Herzog, Andrej Vasilj, Christian Klose, Nicolas Christinat, Delphine Morin-Rivron, Kai Simons, Mojgan Masoodi, Julio L Sampaio

**Affiliations:** 1 Lipotype GmbHDresden, Germany; 2 Nestlé Institute of Health Sciences S.A.Lausanne, Switzerland

**Keywords:** Blood plasma, Clinical biomarker, High throughput, Lipids, Shotgun lipidomics

## Abstract

Blood plasma has gained protagonism in lipidomics studies due to its availability, uncomplicated collection and preparation, and informative readout of physiological status. At the same time, it is also technically challenging to analyze due to its complex lipid composition affected by many factors, which can hamper the throughput and/or lipidomics coverage. To tackle these issues, we developed a comprehensive, high throughput, and quantitative mass spectrometry-based shotgun lipidomics platform for blood plasma lipid analyses. The main hallmarks of this technology are (i) it is comprehensive, covering 22 quantifiable different lipid classes encompassing more than 200 lipid species; (ii) it is amenable to high-throughput, with less than 5 min acquisition time allowing the complete analysis of 200 plasma samples per day; (iii) it achieves absolute quantification, by inclusion of internal standards for every lipid class measured; (iv) it is highly reproducible, achieving an average coefficient of variation of <10% (intra-day), approx. 10% (inter-day), and approx. 15% (inter-site) for most lipid species; (v) it is easily transferable allowing the direct comparison of data acquired in different sites. Moreover, we thoroughly assessed the influence of blood stabilization with different anticoagulants and freeze-thaw cycles to exclude artifacts generated by sample preparation.

**Practical applications:** This shotgun lipidomics platform can be implemented in different laboratories without compromising reproducibility, allowing multi-site studies and inter-laboratory comparisons. This possibility combined with the high-throughput, broad lipidomic coverage and absolute quantification are important aspects for clinical applications and biomarker research.

## Introduction

Lipidomics is the systematic study of pathways and networks of cellular lipids by profiling and understanding the role of lipids in biological systems 1. Blood plasma is widely used in lipidomics studies mainly due to its availability. Furthermore, it carries the lipids incorporated into lipoproteins over the circulatory system from the intestine and liver to all the tissues in the body 2. Because this fluid has access to peripheral tissues, and its lipoproteins exchange lipids with them, its lipid composition should contain a detailed picture of the individual metabolic state 3,4. This makes plasma a prime target for diagnostic research but at the same time very challenging due to its compositional complexity 5.

In order to establish reliable lipid diagnostic biomarkers, one needs to dissect the many factors affecting the plasma lipidome which are not necessarily disease-related, e.g., different genetic backgrounds, diet, gender, age, and life style 6–11. This creates a severe problem and often plasma lipidomics studies are either performed in a high-throughput manner but targeting only a subset of the lipidome 12 or they cover the whole lipidome for a small sample set 5. In lipidomics methodologies, like in other omics techniques, there is an inverse correlation between a method's throughput versus lipidomic structural elucidation and coverage 13. The most comprehensive plasma lipidomic coverage available, offered by the LipidMaps consortium 5, is achieved by the combination of multiple analytical set-ups running in different laboratories 14 which greatly reduces the throughput and feasibility. On the other hand, one-step chromatography-based approaches offer improved throughput at the expense of lower lipidomic coverage 15–19. Nowadays, the available shotgun lipidomics methodologies offer the highest throughput. This is achieved by direct infusion of the extract without previous chromatographic separation with the drawbacks of not providing details about lipid molecular species 20 or limited lipidomic coverage 21.

In this paper, we developed a high throughput mass spectrometry (MS)-based shotgun lipidomics platform that offers quantitative and extended lipidomic coverage down to the molecular lipid species level for systematic lipidomic profiling of large populations. Our platform achieves unprecedented acquisition speed (5 min per acquisition, allowing the analysis of 200 samples per day per MS instrument including sample preparation) combined with high lipidomic coverage (22 quantifiable lipid classes encompassing more than 200 lipid species) in a single acquisition. In order to achieve this performance, we optimized the sample preparation, MS acquisition, and lipid identification approaches. All sample preparation steps were automatized for increased throughput and precision. On the instrumentation side, we took advantage of the configuration of the quadrupole-Orbitrap hardware that allows fast polarity switching, safe precursor selection for MSMS fragmentation analysis, and high mass accuracy and resolution. The combination of these features allows for obtaining multiple complementary MS scans in the same acquisition that enabled us to cover the full spectrum of intact lipids. The increase in sample number and spectral complexity (inclusion of MSMS spectra) greatly inflates the information to be processed. To handle this, we developed a new approach for individual molecular species identification and quantification. Lipid species from high resolution MS spectrum are identified and quantified followed by deconvolution of these lipid species into the different lipid molecular species using the corresponding tandem MS fragments.

In addition to the high coverage and throughput, this technology is also very robust. We observed very small technical variation within the same day, between different days of acquisition and between different acquisition sites. We present, for the first time, a technology so robust that it can be implemented in other laboratories without any compromise in precision. These are important features required in clinical diagnostics screens. Moreover, we assess the impact of sample collection and storage on the plasma lipidome stability.

## Materials and methods

### Chemicals

Water, propan-2-ol, and methanol were purchased from Fischer Scientific. Methyl tert-butyl ether, chloroform, ammonium bicarbonate, and ammonium acetate were purchased from Sigma–Aldrich. All chemicals were analytical grade. Synthetic lipid standards were purchased from Avanti Polar Lipids, Larodan Fine Chemicals, and Sigma–Aldrich.

### Sample collection and preparation

For plasma isolation, blood was collected and centrifuged (2000×*g*, 10 min) and the supernatant was collected 22. Prior to extraction, the plasma was diluted 1:50 v/v by mixing 15 μL of it with 735 μL of 150 mM ammonium bicarbonate aqueous solution, aliquoted, and stored at −80°C. Since we use 1 μL of blood plasma per extraction, this dilution is required to minimize the error arising from handling low volumes of liquid. The whole procedure was finished in 2 h after blood collection. It was used to prepare the reference samples, where plasma from three healthy, unfasted donors was combined in equivolumetric ratios separated in batches according to the anticoagulant used. For all analysis, except the comparison of anticoagulants, the plasma derived from EDTA-stabilized blood samples was used and all samples measured were coming from the same batch of reference samples.

For the anticoagulation comparison, blood was collected into S-Monovette EDTA K3, sodium citrate, and lithium–heparin (all Sarstedt) anticoagulant vacutainers (each donor donated blood to all three containers) according to the producer's manual. The data were corrected for the initial dilution of blood by citrate (blood to citrate 10:1 ratio; v/v).

In the freeze and thaw assessment, the samples were prepared as described above and were thawed at 4°C for 2 h, mixed by vortexing, and frozen by placing back at −80°C for 24 h. One freeze and thaw cycle was performed on a daily basis and immediately after all cycles were completed samples were extracted and analyzed.

### Lipid extraction

The lipid extraction (adapted from Matyash et al. 23) was carried out in high grade polypropylene deep well plates. Fifty microliters of diluted plasma (50×) (equivalent of 1 μL of undiluted plasma) was mixed with 130 μL of ammonium bicarbonate solution and 810 μL of methyl tert-butyl ether/methanol (7:2, v/v) solution was added. Twenty-one microliters of internal standard mixture was pre-mixed with the organic solvents mixture. The internal standard mixture contained: 50 pmol of lysophasphatidylglycerol (LPG) 17:1, 50 pmol of lysophosphatic acid (LPA) 17:0, 500 pmol of phosphatidylcholine (PC) 17:0/17:0, 30 pmol of hexosylceramide (HexCer) 18:1;2/12:0, 50 pmol of phosphatidylserine (PS) 17:0/17:0, 50 pmol of phosphatidylglycerol (PG) 17:0/17:0, 50 pmol of phosphatic acid (PA) 17:0/17:0, 50 pmol of lysophposphatidylinositol (LPI 17:1), 50 pmol of lysophosphatidylserine (LPS) 17:1, 1 nmol cholesterol (Chol) D6, 100 pmol of diacylglycerol (DAG) 17:0/17:0, 50 pmol of triacylglycerol (TAG) 17:0/17:0/17:0, 50 pmol of ceramide (Cer) 18:1;2/17:0, 200 pmol of sphingomyelin (SM) 18:1;2/12:0, 50 pmol of lysophosphatidylcholine (LPC) 12:0, 30 pmol of lysophosphatidylethanolamine (LPE) 17:1, 50 pmol of phosphatidylethanolamine (PE) 17:0/17:0, 100 pmol of cholesterol ester (CE) 20:0, 50 pmol of phosphatidylinositol (PI) 16:0/16:0. The plate was then sealed with a teflon-coated lid, shaken at 4°C for 15 min, and spun down (3000 g, 5 min) to facilitate separation of the liquid phases and clean-up of the upper organic phase. Hundred microliters of the organic phase was transferred to an infusion plate and dried in a speed vacuum concentrator. Dried lipids were re-suspended in 40 μL of 7.5 mM ammonium acetate in chloroform/methanol/propanol (1:2:4, v/v/v) and the wells were sealed with an aluminum foil to avoid evaporation and contamination during infusion. All liquid handling steps were performed using Hamilton STARlet robotic platform with the Anti Droplet Control feature for organic solvents pipetting.

### MS data acquisition

Samples were analyzed by direct infusion in a QExactive mass spectrometer (Thermo Fisher Scientific) equipped with a TriVersa NanoMate ion source (Advion Biosciences). Five microliters were infused with gas pressure and voltage set to 1.25 psi and 0.95 kV, respectively. The delivery time was set to 4 min and 55 s with contact closure delay of 20 s to avoid initial spray instability. Polarity switch from positive to negative mode was set at 135 s after contact closure. Samples were analyzed in both polarities in a single acquisition.

The MS acquisition method starts with positive ion mode by acquiring the *m/z* 402–412 in MS + mode at *R*_*m/z* __= 200_ = 140 000 to monitor the [Chol + NH_4_^+^]^+^ ion for 12 s. All individual scans in every segment are the average of 2 microscans. Automatic gain control (AGC) was set to 5 × 10^5^ and maximum ion injection time (IT) was set to 200 ms. Then we scan the *m/z* 550–1000 in MS + (*R*_*m/z* __= 200_ = 140 000) with lock mass activated at a common background (*m/z* = 680.48022) for 18 s. AGC was set to 10^6^ and IT was set to 50 ms. This is followed by a MSMS + (*R*_*m/z* __= 200_ = 17 500) data independent analysis triggered by an inclusion list for 105 s. The inclusion list contains all the masses from 500.5 to 999.75 with 1 Da intervals. AGC was set to 10^5^ and IT was set to 64 ms. The isolation width was set to 1.0 Da, first mass of MSMS acquisition was 250 Da and normalized collision energy was set to 20%. Both MS+ and MSMS+ data are combined to monitor SE, DAG, and TAG ions as ammonium adducts. After polarity switch to negative ion mode, a lag of 15 s before acquisition was inserted to allow spray stabilization. Then, we scan for the *m/z* 400–650 in FTMS − (*R*_*m/z* __= 200_ = 140 000) for 15 s with lock mass activated at a common background (*m/z* = 529.46262) to monitor LPG, LPA, LPI, LPS, and LPE as deprotonated anions and LPC and LPC O– as acetate adducts. AGC was set to 10^6^ and IT was set to 50 ms. We then scan the *m/z* 520–940 in FTMS − (*R*_*m/z* __= 200_ = 140 000) for 15 s with lock mass activated at a common background (*m/z* = 529.46262). AGC was set to 10^6^ and IT was set to 50 ms. Finally, we scan MSMS- (*R*_*m/z*__= 200_ = 17 500) by data independent analysis triggered by an inclusion list for 90 s. This inclusion list contains all the masses from 590.5 to 939.5 with 1 Da intervals. AGC was set to 10^5^ and IT was set to 64 ms. Isolation width was set to 1.0 Da, first mass of MSMS acquisition was 150 Da, and normalized collision energy was set to 35%. Both MS and MSMS data were combined in order to monitor PC, PC O–, HexCer, Cer, SM as acetate adducts and PS, PG, PA, PE, PE O–, and PI as deprotonated anions.

### Data analysis and post-processing

All data were analyzed with an in-house developed lipid identification software based on LipidXplorer 24,25. All Molecular Fragmentation Query Language queries used in this work are available and can be found in Supplementary Material 1. Tolerance for MS and MSMS identification was set to 2 ppm in scans where we have lock mass activated and 8 ppm when lock mass was not available. Data post-processing and normalization were performed using an in-house developed data management system. Data visualization, linear regression (linear least squares method), and correlation (two-tailed Pearson correlation) calculations were performed on Prism 6.0e software (GraphPad Software, Inc.).

## Results and discussion

### Optimization of sample preparation for automation

Automation is essential in order to handle large number of samples because it increases the speed and throughput of the method, makes it more cost-efficient, and most importantly it improves reproducibility 26 (see section 3.5). To this end, we chose the Hamilton Robotics STARlet system. It allows pipetting of wide volume ranges (1–1000 μL) at high precision with the Anti Droplet Control function limiting organic phase dripping from pipette tips.

Remarkably, most lipidomics studies still use one of almost 60 years old classical extraction protocols at times with minor modifications 27,28. More recently, Matyash et al. 23 introduced a new high throughput oriented extraction procedure where chloroform is replaced by methyltert-butyl ether (MTBE). This extraction achieves high recovery of multiple lipid classes with several important advantages 23. MTBE, compared to chloroform, is non-toxic and non-carcinogenic. The organic lipid-containing phase remains on top of the aqueous phase, reducing the water-soluble contaminants during extract collection. All these factors put together greatly facilitate automated sample handling procedures.

For this study, we defined the optimal time of MTBE-based lipid extraction at which maximum lipid recovery is achieved. To this end, a mixture containing standards representing the most abundant lipid classes was added post-extraction to blood plasma lipids extracted for different times. Surprisingly, we observed that in less than 2 min, equilibrium is reached for all lipid classes tracked (Fig. S1). However, the recovery was more reproducible after 5 min. For this reason, we decided that extracting for 15 min is the best compromise between extraction reproducibility and speed.

### Lipid detection, identification, and quantification

The quadrupole-Orbitrap configuration allows multiple possibilities for lipid identification. Besides the consecutive acquisition in both positive and negative ion mode 20, some lipids can be detected by a top-down approach where the unambiguous identification is possible due to the high resolution of the detector, relying solely on the precursor mass accuracy 29. In order to increase the identification specificity, it can also be combined to a bottom-up approach by introducing fragmentation experiments made possible by the presence of the quadrupole. Moreover, this bottom-up approach allows additional structural elucidation which enables molecular lipid species identification 30,31.

To date, molecular lipid species identification and quantification required multiple rounds of long tandem MS acquisitions in order to obtain the statistics for an accurate measurement. To bypass this and decrease the acquisition time, we propose a new approach that takes advantage of the Q-Orbitrap features. Firstly, from the FTMS high-resolution spectrum, we normalize the de-isotoped (type I and II) intensity of the monoisotopic peak of an endogenous species to the de-isotoped (type I and II) intensity of the monoisotopic peak of the standard of the same class to obtain absolute quantification at the species level:

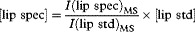
where the concentration of a given lipid species [lip spec] is given by the ratio of its de-isotoped monoisotopic intensity (*I*(lip spec)_MS_) to the added lipid standard de-isotoped monoisotopic intensity (*I*(lip std)_MS_) multiplied by the concentration of the lipid standard [lip std]. After quantification at the species level, the amounts of all *x* molecular phospholipid species with overlapping sum compositions, can then be deconvoluted from the *n* acyl anions fragmentation information contained in the tandem MS data, as follows:

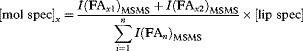
where the concentration of a given molecular species *x*, [mol spec]_*x*_, is given by the sum of the intensities of its complementary acyl anions FA*_x_*_1_ and FA*_x_*_2_, divided by the sum of the intensities of *n* acyl anions from all other isobaric lipid molecular species present in the same MSMS scan, which corresponds to the molar fraction of each lipid molecular species. With this approach, we circumvent the need for multiple cycles of MSMS in order to achieve accurate quantification greatly reducing the time of acquisition.

Phospholipids display variable but in general efficient fragmentation that allows the elucidation of the fatty acid composition 31, but other lipid classes can be more difficult to assess. For example, because triacylglycerides contain combinations of three fatty acids, this makes the assignment of molecular lipid species without chromatographic separation and MS^n^ type of experiments virtually impossible 32. Also, the fragmentation of sphingolipids is very inefficient and not suitable for structure elucidation. The most notable case is sphingomyelin. It presents only one abundant phosphocholine fragment in positive mode that does not give any additional structural information. Conversely, in negative ion mode there is a fragment corresponding to the loss of the amide-linked fatty acid allowing the molecular lipid species elucidation but it cannot be used for identification due to its low intensity. In Table 1, we present a strategy to identify each lipid class and the level of structural detail that can be achieved.

**Table 1:** Mode of acquisition and identification (see section 2.4 for details), structural detail, optimal sample amounts and their *r*^*2*^, dynamic range, its quantification slopes and their *r*^*2*^, LOQ for every lipid class (see Figs. S1 and S3 for additional information)

Lipid class	Mode of identification	Structural detail	Sample amount linear range (uL sample)/linearity (*r*^2^)	Dynamic range (μM)	Slope/Linearity (*r*^2^)
CER	Neg FTMS	Species	0.2–20/0.9997	0.05–250	0.9776/0.9980
CHOL	Pos FTMS	Molecular species	0.2–20/0.9935	20–5000	1.045/0.9969
DAG	Pos FTMS + MSMS	Molecular species	0.2–20/0.9968	0.5–500	0.9271/0.9995
HEXCER	Neg FTMS	Species	–b	1.5–150	0.9636/0.9953
LPA	Neg FTMS	Molecular species	–b	0.25–250	0.9874/0.9990
LPC, LPC O–	Neg FTMS	Molecular species	1.0–20/0.9860	2.5–250	1.017/0.9999
LPE, LPE O–	Neg FTMS	Molecular species	0.2–20/0.9925	0.15–150	0.9818/0.9980
LPI	Neg FTMS	Molecular species	1.0–20/0.9992	0.25–250	0.9794/0.9975
LPS	Neg FTMS	Molecular species	–b	1–250	0.9831/0.9977
PA	Neg FTMS + MSMS	Molecular species	–b	1–250	1.022/0.9967
PC	Neg FTMS + MSMS	Molecular species	0.2–2.0/0.9998	5–2500	1.101/0.9919
PC O–	Neg FTMS	Species	0.2–2.0/1.0000	5–2500	1.101/0.9919
PE	Neg FTMS + MSMS	Molecular species	0.2–20/0.9980	1–250	1.035/0.9981
PE O–	Neg FTMS	Species	0.2–20/0.9988	1–250	1.035/0.9981
PG	Neg FTMS + MSMS	Molecular species	–b	0.25–250	0.9467/0.9953
PI	Neg FTMS + MSMS	Molecular species	0.2–20/0.9965	0.25–250	0.9498/0.9997
PS	Neg FTMS + MSMS	Molecular species	–b	0.25–250	0.9460/0.9973
CE	Pos FTMS + MSMS	Molecular species	0.2–20/0.9976	2–500	0.9001/0.9969
SM	Neg FTMS	Species	0.2–20/0.9936	0.4–1000	0.9554/0.9970
TAG	Pos FTMS + MSMS^a^	Species	0.2–20/0.9965	1–250	1.025/0.9832

a Although MSMS was used to increase the confidence of identification, we could not assign molecular species for the TAG (see text for details).

b Not measurable in this reference sample.

### Optimal sample amount determination for blood plasma lipid extraction

One of the biggest challenges of comprehensive lipid analysis is the huge dynamic range of lipid abundances observed in biological samples like blood plasma. Chromatographic techniques minimize the ion suppression effect of highly abundant lipid species by resolving the different lipids in a temporal dimension 33. This allows for extracting a wide range of sample amounts that to some extent can be tuned for the detection of low abundant species.

In a shotgun lipidomics approach, sample infusion is continuous and the acquisition is done simultaneously for all the different lipid classes, which makes it more susceptible to ion suppression 34. To minimize this, the fine tuning of the sample amount to be extracted is of paramount importance for shotgun-based experiments 21. We extracted different sample amounts together with fixed amounts of internal standards in order to determine the minimum and maximum amounts that give reliable lipid compositions. We observed that most lipid classes give a linear response over two orders of magnitude of sample amount (0.2–20 μL) (Fig. S2). Importantly, we observe a consensual sample range (1–2 μL), which provides a satisfying compromise between lipidomic coverage and sensitivity, and where reproducibility is the highest (Table1 and Fig. S1). It is important to note that this amount is optimized for healthy individuals and needs to be carefully assessed in metabolically challenged conditions where the concentration of specific lipid classes such as triglycerides in blood can increase significantly. This sample amount corresponds to a final sample dilution in the infusate of 1/320 to 1/160 which is in agreement with a previous study for the optimal sample amount for shotgun lipidomics experiments 21.

For quantification, we used one internal standard per lipid class. These internal standards were chosen based on two criteria: first, its absence in plasma samples and second, its similarity to the structure and properties (e.g., extraction recovery and ionization efficiency) of the analytes. This approach is valid due to the fact that the recovery and ionization efficiency of different lipid species within a lipid class is predominantly dependent on their charged head group while their differential acyl chains only minimally alter their ionization at low lipid concentrations 35,36 (Fig. S3). Two notable exceptions are present in the SM profile. SM 42:1;2 apparent relative decrease with sample amount increase is explained by a small background peak of a similar overlapping mass observed already in the blank samples. Importantly, this effect is only significant at very low sample amounts and its impact at the optimal sample amount is minimal. For SM 34:1;2, we observed a relative increase up to 15% in its proportion across the two orders of magnitude of sample amount. Although this is a significant difference, it is not observed in other SM species. This effect is most likely related to differential matrix effects introduced by variable amounts of plasma sample extracted and its impact is irrelevant when we fix the sample amount.

In conclusion, we obtain identical lipid species profiles at different sample concentrations, confirming the assumption that ionization within a lipid class is not lipid species specific for the sample amounts tested allowing to express the quantities in molar amounts. This observation allows us to conclude that, for the optimal sample amount, all lipid species measured within a lipid class have similar response factors enabling absolute quantification with a single standard addition per lipid class.

### Method dynamic range and limit of quantification

Different lipid classes exhibit different extraction, ionization, and fragmentation efficiencies, so the limit of quantification (LOQ) and dynamic ranges are lipid class-specific. In order to determine all these parameters, we added different amounts of each lipid class standard to the fixed optimal plasma sample amount (1 μL) and we recorded how the signal acquired correlates with the standard amount added. The LOQ was defined by the minimum concentration at which the slope and the correlation coefficient (*r*) are not significantly compromised and the variation of the standard intensity is below 20%. The summary of the results obtained can be found in Table1 and Fig. S3. We observed that the lipid classes showing a higher LOQ (>1 µM) correspond to abundant lipid classes in plasma (e.g., Chol, SE, and TAG); therefore, a higher detection limit should not have a significant impact on their species. Interestingly, they are all acquired in positive ion mode as an ammonium adduct, so an increase in the ammonium acetate concentration in the MS-infusion mixture can potentially improve their detection sensitivity, if required. On the other hand, anionic lipids that are usually acquired in negative ion mode, exhibit LOQs down to 50 nM. We conclude that the absence or low number of lipid species identified in some classes such as HexCer, LPA, LPS, and PA is due to their low abundance in this particular sample rather than low sensitivity for these lipid classes.

### Assessment of the method reproducibility

Reproducibility is the ability to obtain similar results by the same researcher on different days and/or by someone else working independently. In this work, we aimed at maximizing method reproducibility by automation of most of the sample preparation, extraction, data acquisition and, to some extent, lipid identification, and post-processing of the data.

In order to make a thorough assessment of the reproducibility of our method, we performed a systematic series of experiments where we aliquoted the same reference sample 540 times across six independent 96-well plates. Three of these plates (270 aliquots in total) were subjected on three different days (90 aliquots per day) to extraction and analysis in one laboratory and the other three plates were processed similarly in another laboratory. Both sites used identical platforms for the sample processing and analysis. Since every sample is an aliquot of the same batch of plasma (see section 2.2), the variation observed across different acquisitions, accurately reflects the intra-plate variation (variation within the same day of acquisition), inter-day variation (variation on different days of acquisition), and inter-site variation (variation between different sites).

**Figure 1:**
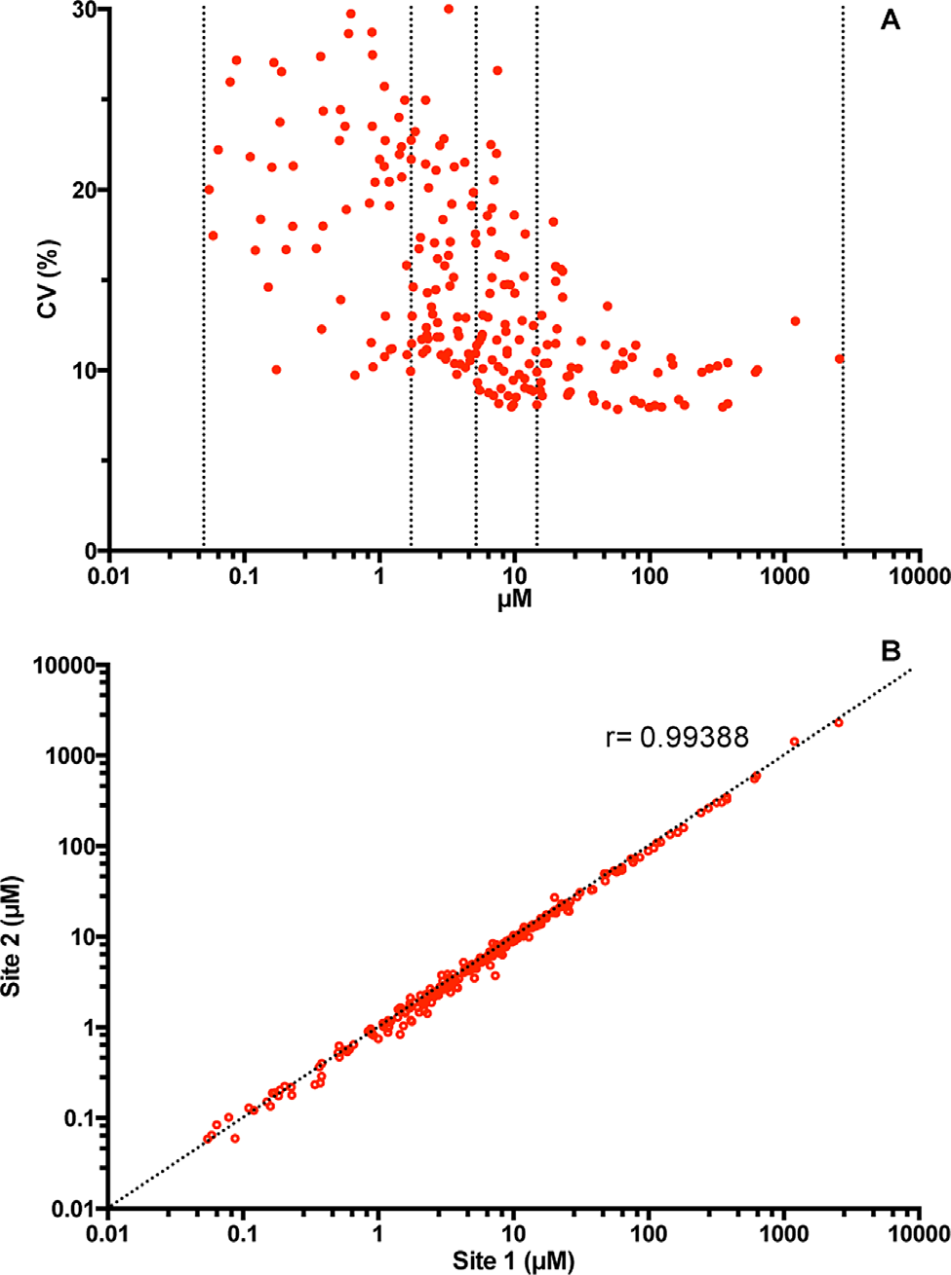
(A) Correlation between coefficients of variation and average lipid concentrations from 270 individual measurements performed on three different days. The dashed lines separate the quartiles according to lipid concentration (see Table2 for additional information). (B) Pearson correlation plot of averaged concentrations determined at the two sites. Every point represents the average concentration of lipid species measured from 270 independent acquisitions. Correlation coefficient (*r*) is given.

It is important to note that in analytical methods, there is usually an inverse correlation between lipid amount present in the sample and the coefficient of variation 21. Because different lipid species can range in amounts by several orders of magnitude, one has to assess how the amount affects the reproducibility of a given molecular lipid species. To assess method variation, we sorted the lipids by abundance and divided the data in quartiles and averaged the coefficient of variation within each quartile (Fig. 1A and Table2).

**Table 2:** Averaged coefficients of variation within the different lipid concentration quartiles obtained on the same day (intra-plate), on different days (inter-day), and in different sites (inter-site) (see Fig. 1 for additional information)

				Variation analysis		
Quartile	Range of concentrations (μM)	No. of species	Coverage (mol%)	Intra-plate CV (*n* = 3)	Inter-day CV	Inter-site CV
First	16–2500	56	93.1	6.6 ± 1.1	10.5	11.6
Second	5–16	56	4.8	9.5 ± 0.91	12.8	14.6
Third	1.5–5	56	1.7	12.3 ± 1.0	15.5	18.7
Fourth	0.05–1.5	56	0.4	16.8 ± 0.2	19.8	22.3

We observed that the coefficient of variation indeed correlates inversely with the lipid amount but importantly we still obtain low CVs when the plates are processed on different days and in different laboratories (Table2 and Fig. 1B). It is important to note that, although the first and second quartiles correspond to half the number of lipid species analyzed, when taking in consideration their amounts, they correspond to 98% of the lipidome and they can be measured with CVs lower than 15% on average even when acquired at different sites. For the third and fourth quartiles, the least abundant species, we can also conclude that the higher CV can be attributed to their intrinsic low abundance and not to any irreproducibility bias.

### Effect of anticoagulants and freeze-thaw cycles on blood plasma lipidomes

**Figure 2:**
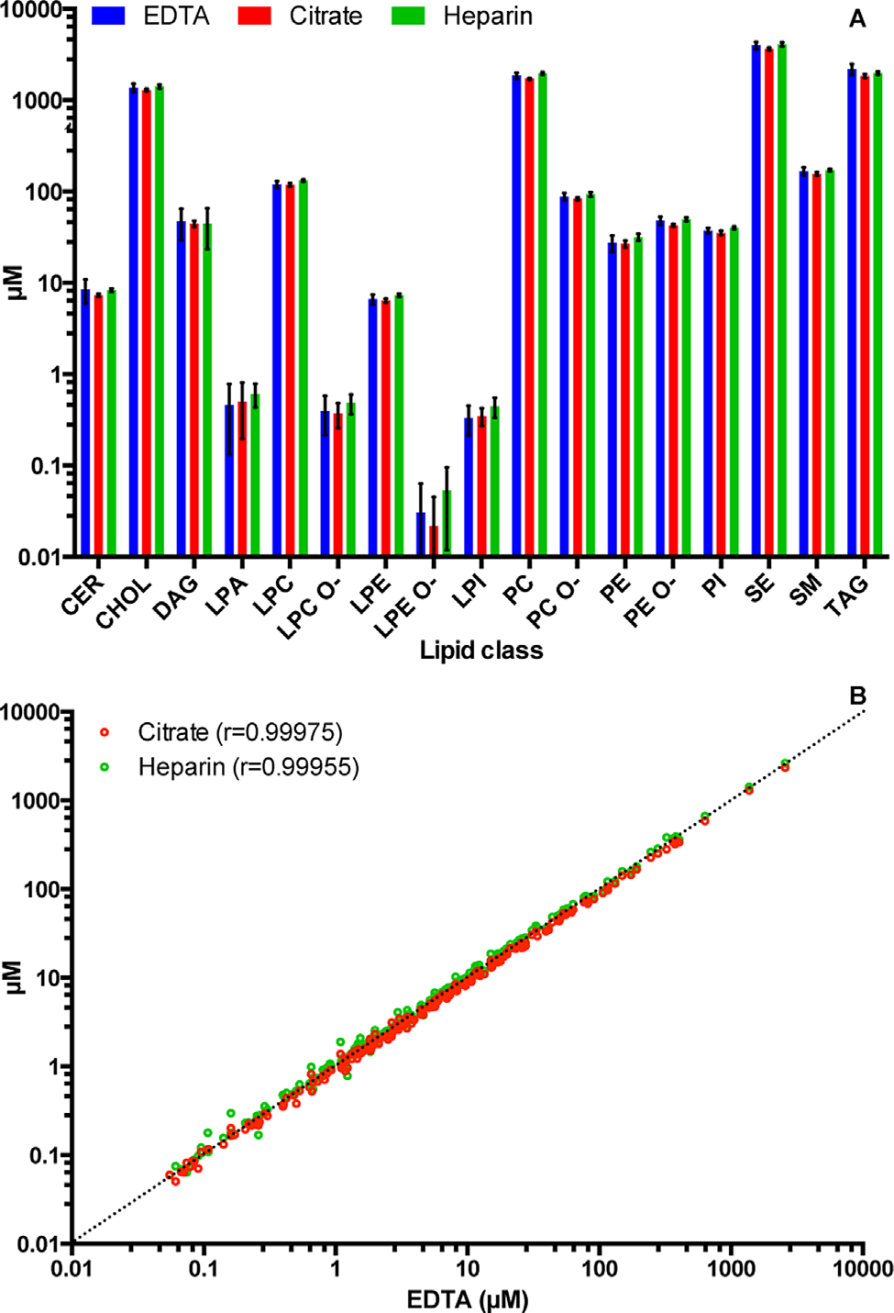
Effect of anticoagulants on the blood plasma lipidome analysis. (A) Lipid class profile comparison of plasma collected with EDTA, citrate, and heparin as anticoagulants. Averaged values are shown with error bars depicting standard deviation of 27 independent experiments for each anticoagulant. (B) Pearson correlation of the lipids species quantified. Every point represents the average concentration of lipid species calculated from 27 independent experiments for each condition. Correlation coefficients (*r*) versus EDTA are given.

From the sample collection to spectral acquisition, there are several steps that might affect the state of the sample and as a consequence alter the final result. During the sample collection, it is necessary to add anticoagulants to the blood sample when plasma preparation is considered. However, throughout the literature, different anticoagulants have been used indiscriminately without any verification of the impact that they might have on the lipid analysis, though it is known that they can influence multiple parameters of blood 37. To assess if different anticoagulants used for blood stabilization affected the plasma lipidome, we compared plasma samples derived from blood stabilized with the three most commonly used substances: EDTA, citrate, and heparin. All samples, regardless of the anticoagulant used, yielded remarkably comparable lipidomes, with Pearson correlation coefficients exceeding 0.999 and had almost identical lipid class profiles (Fig. 2). Interestingly, we observed that citrate containing samples display systematically lower lipid amounts when compared to samples containing other anticoagulants. This is due to the fact that citrate in vacutainers is present as an aqueous solution and is mixed with the blood during drawing, therefore, diluting it. Although we corrected for the dilution to the best of our ability, we did not manage to retrieve results identical to the other anticoagulants. This has been observed previously 38, which makes us conclude that citrate should be avoided if possible due to the introduction of uncertainty in the sample volume determination.

**Figure 3:**
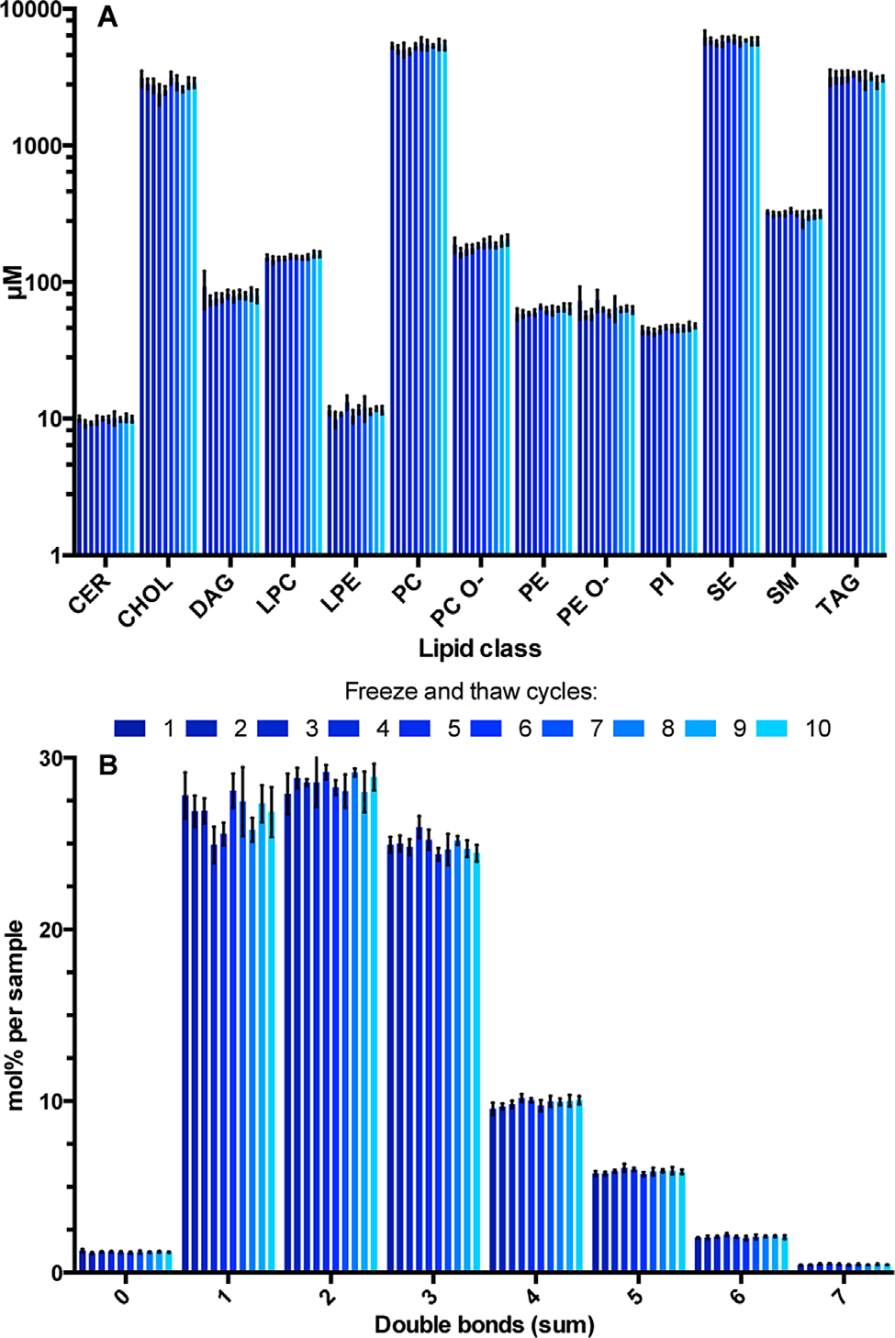
Effect of the number of freeze and thaw cycles on the plasma lipidome. (A) Lipid class profile of the same sample frozen and thawed up to 10 times. (B) Double bond profile of the same sample frozen and thawed up to 10 times. Averaged values are shown with error bars depicting standard deviation of five experiments per freeze and thaw cycle.

In a given study, it might be useful to re-analyze the same sample in order to obtain additional information. As it is often assumed, freeze-thawing cycles might influence sample and analyte properties: in this particular case, the lipids. We evaluated how freezing and thawing affected the plasma lipidome by performing 10 freeze-thaw cycles prior to extraction. We did not detect any significant systematic alterations in lipid levels, nor in molecular species composition of lipid classes that could be correlated to the number of freeze and thaw cycles (Fig. 3A). More importantly, we did not observe any decrease in the unsaturation level of fatty acids present in lipids, which would be a sign of oxidation of polyunsaturated fatty acids (Fig. 3B). These results corroborate previous observations 39,40. It is worth to mention that the definition of the maximum number of freeze and thaw cycles without sample damage is still controversial 41. We believe that the controversy might be related more to the specific conditions of freeze-thaw cycles in different studies than to the number of cycles performed. With this in mind, instead of claiming that one can perform 10 freeze and thaw cycles without sample degradation, we would rather emphasize that thawing the samples at 4°C as quickly as possible, should allow for at least 10 cycles without sample degradation as has been shown in this study. Taken together, these results provide guidelines for consistent sample collection, storage, and re-acquisition. However, although the effects of sample collection and freeze-thawing on the plasma lipidome were ruled out, other factors leading to degradation of lipids cannot be excluded. For instance, it is known that physical stress imposed on leukocytes during blood drawing may activate them (especially when hemolysis occurs that might take place in blood from anemic patients) and induce the arachidonic acid cascade, which in turn can influence the plasma lipidome 5. However, as these effects potentially appear during sample collection, their influence on the measured lipidome is beyond the reach of the methodology described here.

## Conclusions and future perspectives

**Figure 4:**
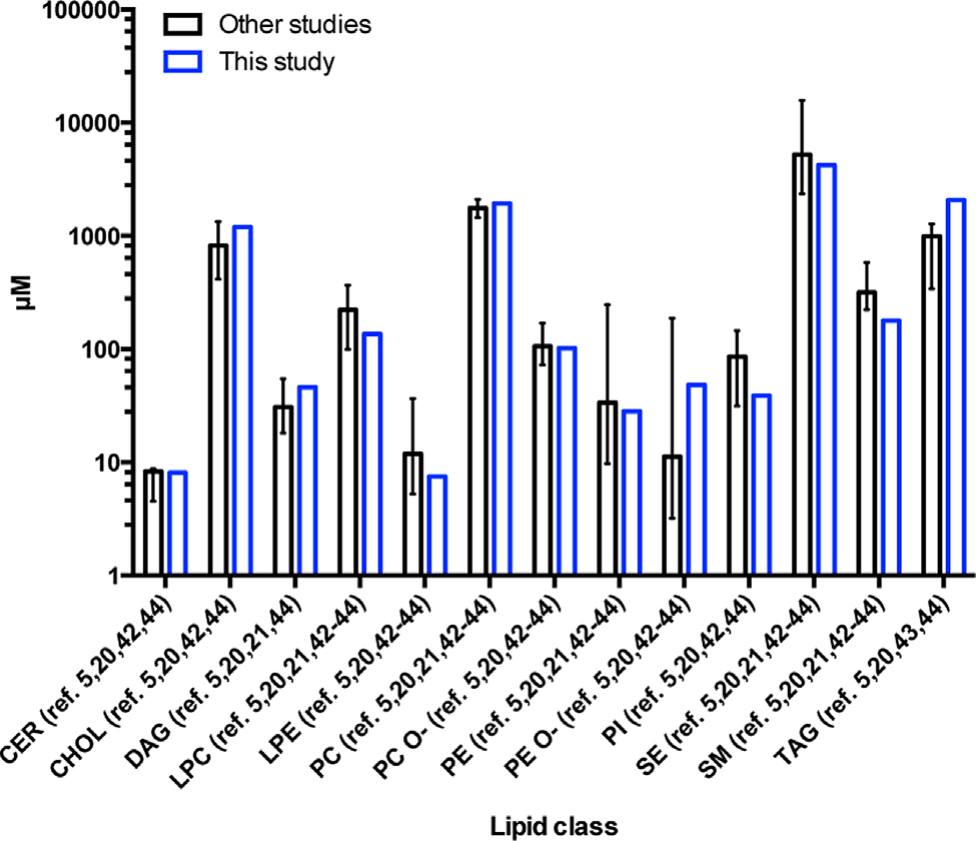
The main lipid classes’ amount distribution described in literature 5,20,21,42–44 compared with this study. Medians of average for control samples reported in these papers are presented. Error bars denote minimal and maximal values (range). Note that not all classes were analyzed in every study.

We have established a fully automated, high throughput shotgun-based lipidomics method for systematic screening of plasma samples. Currently, this is the most comprehensive MS-based lipidomics method from a single acquisition, and most importantly, it is quantitative and highly reproducible. More important than speed and reproducibility is accuracy. We sampled the literature in order to compare our data with the results obtained in studies providing quantitative data and similar lipidomic coverage to ours 5,20,21,42–44. We observed that our data fit the range of values observed in literature, with the exception of TAG and SM (Figs. 4 and S5). These discrepancies are most likely related to the increase in chylomicron content in the plasma that we used from unfasted donors. Chylomicrons are known to transport lipids from the intestine and an increase in TAG content should be expected in unfasted subjects, since they are the most TAG enriched lipoproteins particles present in plasma 45.

To the best of our knowledge, for the first time for an omics-type analytical technology, we showed high method precision not only on different days but also in different laboratories. The implications of these are twofold. First, it means that this technology can be easily implemented in different sites without compromising data reproducibility and second, it facilitates direct integration of data between different laboratories, allowing multi-site studies for higher throughput, if required.

We believe this method paves the way for making lipidomics an accessible and indispensable tool not only in biological basic research, but also for clinical diagnostics and nutrition by offering unprecedented throughput and accuracy.

The authors would like to thank to Andrej Shevchenko for feedback on the paper. This study was supported by Nestlé Institute of Health Sciences and the Klaus Tschira Foundation.

Conflict of interest statement: MAS, RH, AV, CK, KS, JLS have paid employment at Lipotype GmbH and NC, DM-R, MM have paid employment at Nestlé Institute of Health Sciences S.A. This does not alter the authors’ adherence to all policies on sharing data and materials.

## Abbreviations

AGC: automatic gain control
IT: injection time
LOQ: limit of quantification
MS: mass spectrometry
MTBE: methyl-tert-butyl ether

## Supporting Information

As a service to our authors and readers, this journal provides supporting information supplied by the authors. Such materials are peer reviewed and may be re-organized for online delivery, but are not copy-edited or typeset. Technical support issues arising from supporting information (other than missing files) should be addressed to the authors.

**Figure S1:** Lipid extraction kinetics for different lipid classes.

**Figure S2:** Sample amount titration.

**Figure S3:** Effect of sample amount on the normalized lipid species profile per lipid class.

**Figure S4:** Dynamic range determination per lipid class.

**Figure S5:** The main lipid species’ normalized distribution described in plasma lipidomics literature[5,20,21,42-44] compared with this study.

Table S1.

Figure Caprion
