# Supplementary figures and images for: An automated shotgun lipidomics platform for high throughput, comprehensive, and quantitative analysis of blood plasma intact lipids
Source: Eur J Lipid Sci Technol. 2015 Jul 20;117(10):1540–9. doi: 10.1002/ejlt.201500145 (PMC4606567; doi:10.1002/ejlt.201500145)

CER

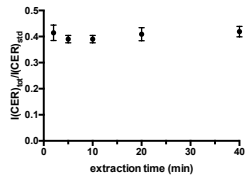

PE O-

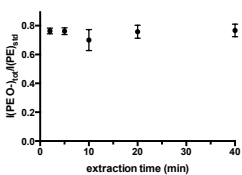

LPI

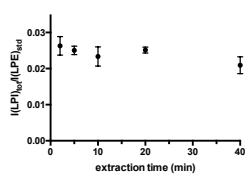

PE

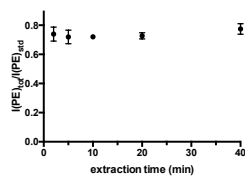

LPE

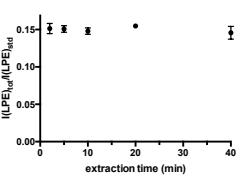

PC O-

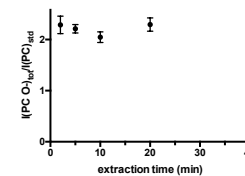

LPC

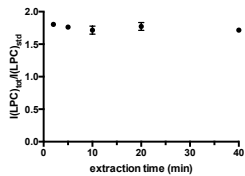

PC

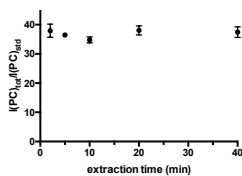

SM

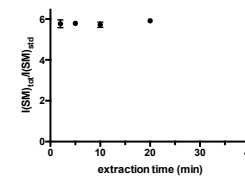

DAG

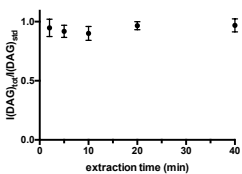

PI

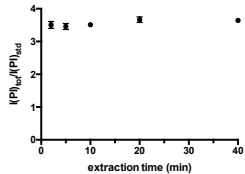

SE

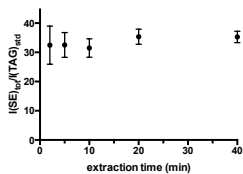

TAG

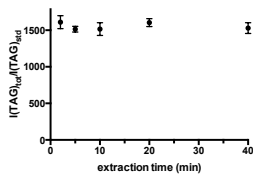

Supplement: Supplementary file 1 — Figure S1: Lipid extraction kinetics for different lipid classes. [file ejlt0117-1540-sd1.pdf]

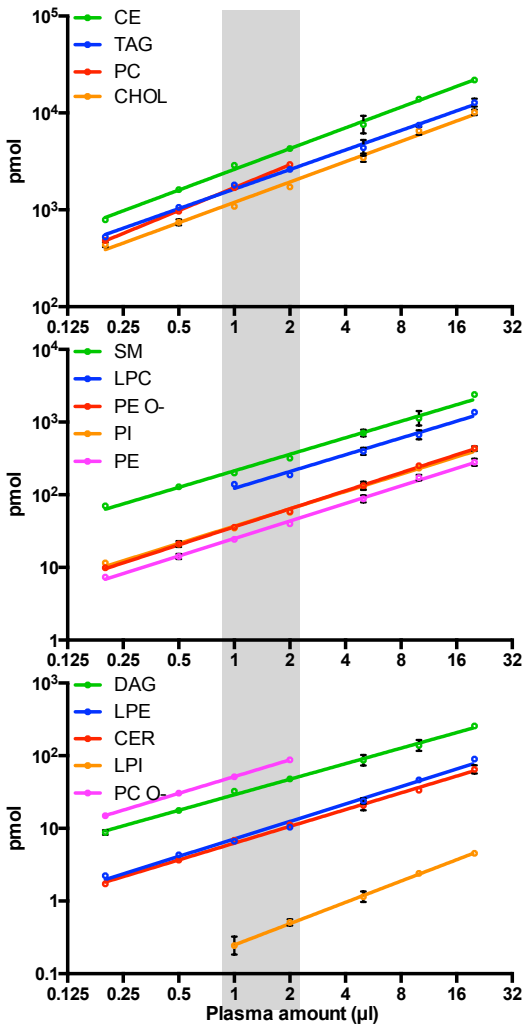

Supplement: Supplementary file 2 — Figure S2: Sample amount titration. [file ejlt0117-1540-sd2.pdf]

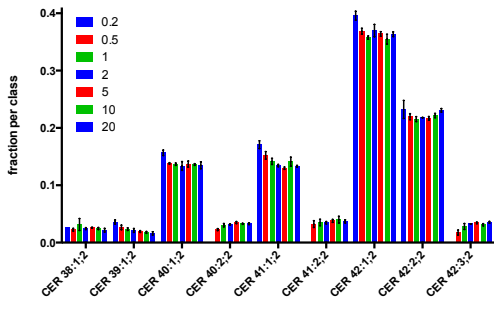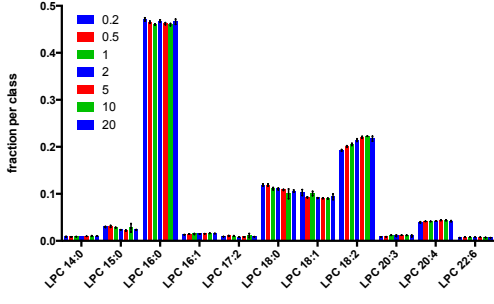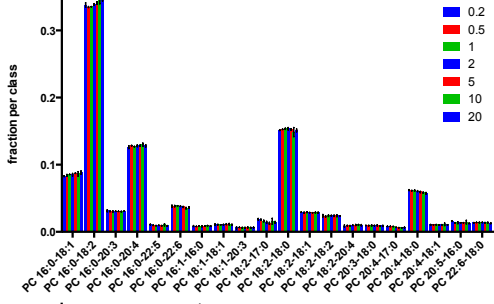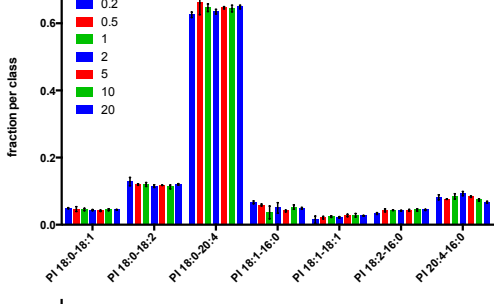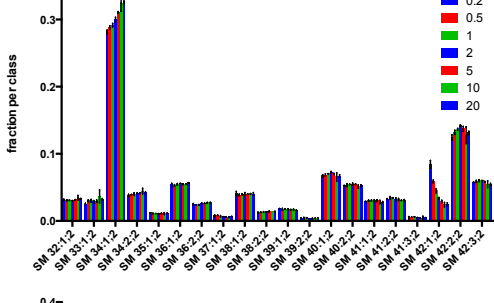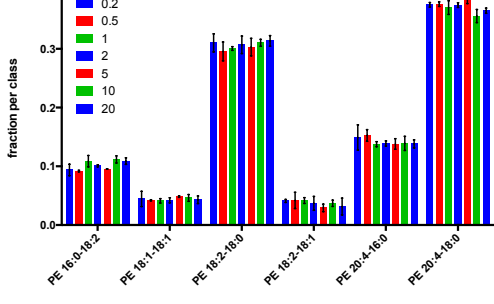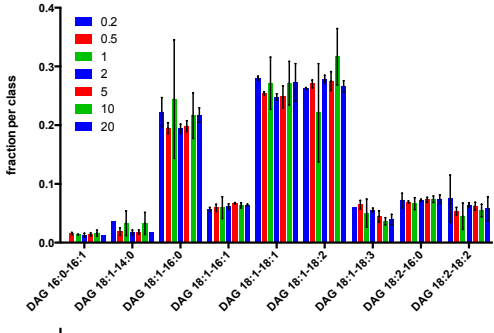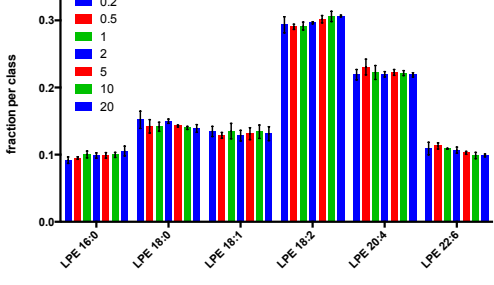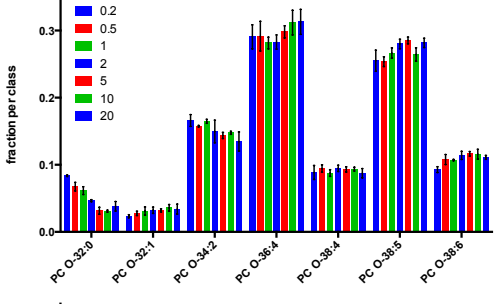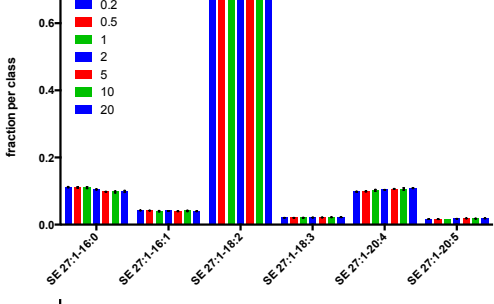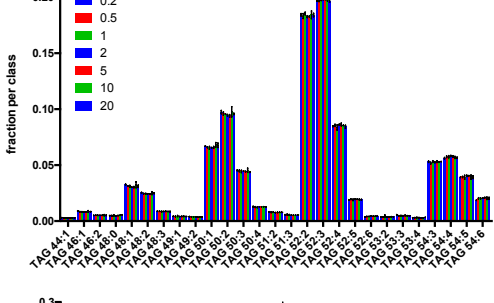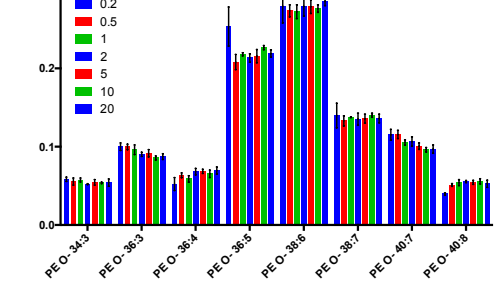

Supplement: Supplementary file 3 — Figure S3: Effect of sample amount on the normalized lipid species profile per lipid class. [file ejlt0117-1540-sd3.pdf]

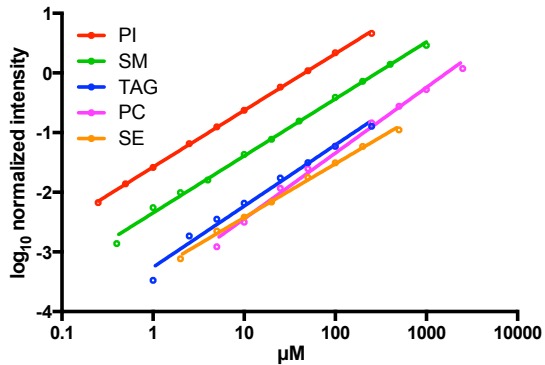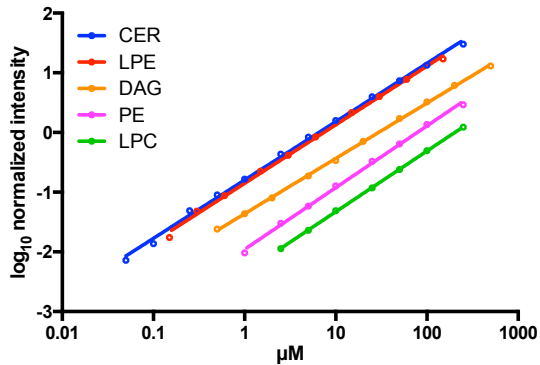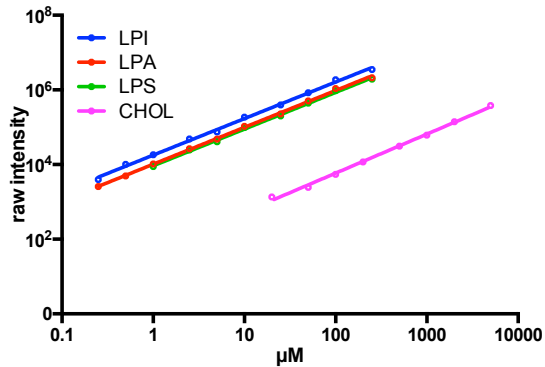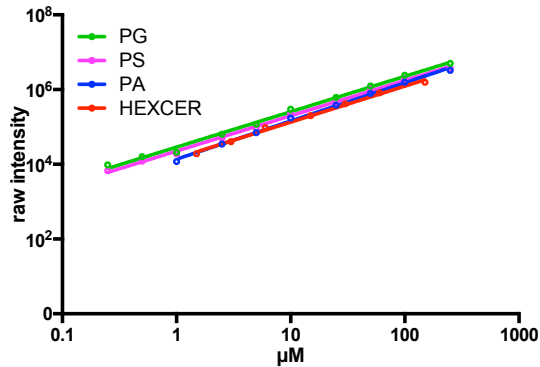

Supplement: Supplementary file 4 — Figure S4: Dynamic range determination per lipid class. [file ejlt0117-1540-sd4.pdf]
