# Supplementary material for: An automated shotgun lipidomics platform for high throughput, comprehensive, and quantitative analysis of blood plasma intact lipids
Source: Eur J Lipid Sci Technol. 2015 Jul 20;117(10):1540–9. doi: 10.1002/ejlt.201500145 (PMC4606567; doi:10.1002/ejlt.201500145)
Supplement: Supplementary file 7 — Figure Caprion [file ejlt0117-1540-sd7.doc]

**An Automated Shotgun Lipidomics platform for high throughput comprehensive analysis of blood plasma Intact Lipids**

Michal A. Surma†, Ronny Herzog†, Andrej Vasilj†, Christian Klose†, Nicolas Christinat‡, Delphine Morin-Rivron‡, Kai Simons†, Mojgan Masoodi‡*, Julio L. Sampaio†*

† Lipotype GmbH, Tatzberg 47, 01307 Dresden, Germany

‡ Nestlé Institute of Health Sciences S.A., EPFL Innovation Park Bâtiment H1015, Lausanne, Switzerland

* Corresponding authors:

[sampaio@lipotype.de](mailto:sampaio@lipotype.de) Tel:+49-351-796 5343;

[mojgan.masoodi@rd.nestle.com](mailto:mojgan.masoodi@rd.nestle.com) Tel:+41-21-6326156

**Table of contents:**

**Figure S-1.** Lipid extraction kinetics for different lipid classes.

**Figure S-2.** Sample amount titration.

**Figure S-3.** Effect of sample amount on the normalized lipid species profile per lipid class.

**Figure S-4.** Dynamic range determination per lipid class.

**Figure S-5:** The main lipid species’ normalized distribution described in plasma lipidomics literature[5,20,21,42–44] compared with this study.

**Figure S-1.** Lipid extraction kinetics for different lipid classes. Data points correspond to the average of 4 independent lipid extractions. Error bars correspond to the standard deviation of the measurements.

**Figure S-2.** Sample amount titration. Only ranges with linear behaviour are shown. Data points correspond to the average of 3 independent experiments. Error bars correspond to the standard deviation. A summary of the data can be found in Table 1.

**Figure S-3.** Effect of sample amount on the normalized lipid species profile per lipid class. Data points correspond to the average of 3 independent experiments. Error bars correspond to the standard deviation.

**Figure S-4.** Dynamic range determination per lipid class. Internal standards were spiked in the plasma sample (at the optimal sample amount) and the intensity recorded (see Materials and Methods for additional information). Data points correspond to the average of 3 independent experiments. Error bars correspond to the standard deviation. A summary of the data can be found in Table 1.

**Figure S-5:** The main lipid species’ normalized distribution described in plasma lipidomics literature[5,20,21,42–44] compared with this study. Medians of average for control samples reported in these studies are presented. Error bars denote minimal and maximal values (range).
