# Supplementary material for: An automated shotgun lipidomics platform for high throughput, comprehensive, and quantitative analysis of blood plasma intact lipids
Source: Eur J Lipid Sci Technol. 2015 Jul 20;117(10):1540–9. doi: 10.1002/ejlt.201500145 (PMC4606567; doi:10.1002/ejlt.201500145)
Supplement: Supplementary file 5 — Figure S5: The main lipid species’ normalized distribution described in plasma lipidomics literature[5,20,21,42-44] compared with this study. [file ejlt0117-1540-sd5.pdf]

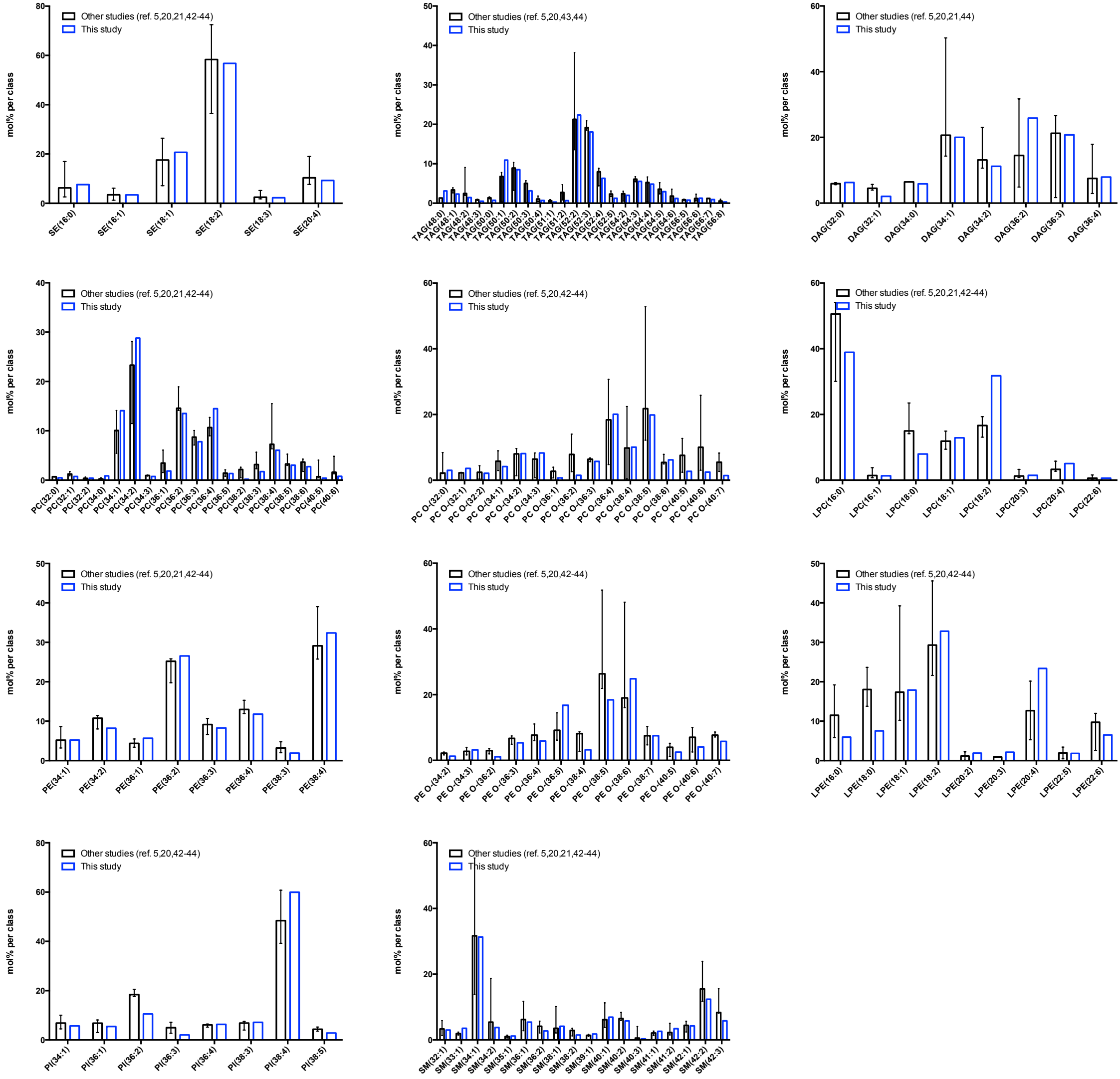

Other studies (ref. 5,20,21,42-44)
This study
mol% per class
SE(16:0) SE(16:1) SE(18:1) SE(18:2) SE(18:3) SE(20:4)
Other studies (ref. 5,20,43,44)
This study
mol% per class
TAG(48:0) TAG(48:1) TAG(48:2) TAG(48:3) TAG(50:0) TAG(50:1) TAG(50:2) TAG(50:3) TAG(50:4) TAG(51:1) TAG(51:2) TAG(52:2) TAG(52:3) TAG(52:4) TAG(52:5) TAG(54:2) TAG(54:3) TAG(54:4) TAG(54:5) TAG(54:6) TAG(56:5) TAG(56:6) TAG(56:7) TAG(56:8)
Other studies (ref. 5,20,21,44)
This study
mol% per class
DAG(32:0) DAG(32:1) DAG(34:0) DAG(34:1) DAG(34:2) DAG(36:2) DAG(36:3) DAG(36:4)
Other studies (ref. 5,20,21,42-44)
This study
mol% per class
PC(32:0) PC(32:1) PC(32:2) PC(34:0) PC(34:1) PC(34:2) PC(34:3) PC(36:1) PC(36:2) PC(36:3) PC(36:4) PC(36:5) PC(38:2) PC(38:3) PC(38:4) PC(38:5) PC(38:6) PC(40:5) PC(40:6)
Other studies (ref. 5,20,42-44)
This study
mol% per class
PC O-(32:0) PC O-(32:1) PC O-(32:2) PC O-(34:1) PC O-(34:2) PC O-(34:3) PC O-(36:1) PC O-(36:2) PC O-(36:3) PC O-(36:4) PC O-(38:4) PC O-(38:5) PC O-(38:6) PC O-(40:5) PC O-(40:6) PC O-(40:7)
Other studies (ref. 5,20,21,42-44)
This study
mol% per class
LPC(16:0) LPC(16:1) LPC(18:0) LPC(18:1) LPC(18:2) LPC(20:3) LPC(20:4) LPC(22:6)
Other studies (ref. 5,20,21,42-44)
This study
mol% per class
PE(34:1) PE(34:2) PE(36:1) PE(36:2) PE(36:3) PE(36:4) PE(38:3) PE(38:4)
Other studies (ref. 5,20,42-44)
This study
mol% per class
PE O-(34:2) PE O-(34:3) PE O-(36:2) PE O-(36:3) PE O-(36:4) PE O-(36:5) PE O-(38:4) PE O-(38:5) PE O-(38:6) PE O-(38:7) PE O-(40:5) PE O-(40:6) PE O-(40:7)
Other studies (ref. 5,20,42-44)
This study
mol% per class
LPE(16:0) LPE(18:0) LPE(18:1) LPE(18:2) LPE(20:2) LPE(20:3) LPE(20:4) LPE(22:5) LPE(22:6)
Other studies (ref. 5,20,42-44)
This study
mol% per class
PI(34:1) PI(36:1) PI(36:2) PI(36:3) PI(36:4) PI(38:3) PI(38:4) PI(38:5)
Other studies (ref. 5,20,21,42-44)
This study
mol% per class
SM(32:1) SM(33:1) SM(34:1) SM(34:2) SM(35:1) SM(36:1) SM(36:2) SM(38:1) SM(38:2) SM(39:1) SM(40:1) SM(40:2) SM(40:3) SM(41:1) SM(41:2) SM(42:1) SM(42:2) SM(42:3)
